# Psychometric evaluation of patient assessment of chronic illness care among Korean cancer survivors

**DOI:** 10.1371/journal.pone.0256119

**Published:** 2021-08-12

**Authors:** Soo Hyun Kim, Bo Gyeong Lee, Yu Hyeon Choe

**Affiliations:** 1 Department of Nursing, Inha University, Incheon, South Korea; 2 College of Nursing, Research Institute of Nursing Science, Daegu Catholic University, Daegu, South Korea; Universita degli Studi di Firenze, ITALY

## Abstract

**Background:**

The Patient Assessment of Chronic Illness Care (PACIC) was developed in the United States to assess the implementation of the Chronic Care Model (CCM)-based intervention from the patient’s perspective. Although the psychometric properties of the PACIC have been reported in other chronically ill patients, it has not been reported in cancer survivors. Our aim was to evaluate the acceptability, validity, and reliability of a Korean version of the PACIC among cancer survivors (K-PACIC-CS).

**Methods:**

Among 204 cancer survivors at a university-based hospital in South Korea, we performed psychometric evaluation of the K-PACIC-CS according to acceptability (descriptive statistics, missing values, and floor and ceiling effects), validity (confirmative factor analysis [CFA] and convergent validity), and reliability (internal consistency, i.e., Cronbach’s alpha).

**Results:**

The item response was high (missing rate = 0.5%). The floor effect was 3.9%– 43.6% and the ceiling effect was 6.9%– 41.2%. The CFA revealed good indices of fit and confirmed the five structures predetermined in the original version of PACIC. The K-PACIC-CS scores had significant positive relationships with cancer survivors’ self-efficacy and health-related quality of life. The total K-PACIC-CS showed excellent internal consistency (Cronbach’s alpha = .94) and those of the subscales were acceptable (Cronbach’s alpha = .76 -.86).

**Conclusions:**

This study suggests that the K-PACIC-CS is a valid and reliable instrument for measuring implementation of CCM-based chronic care from the survivor’s perspective.

## Introduction

Owing to advances in early detection of cancer and its treatment, the number of cancer survivors has increased remarkably. There were more than 16.9 million people with history of cancer in United States (US) in 2019, and that number is projected to reach more than 22.1 million by 2030 [1]and more than 1,800,000 in South Korea in 2019 [2]. To date, approximately 50% of patients diagnosed with cancer survive for 10 years or more [1,3], and that is likely to have a substantial impact on the healthcare system.

Several health challenges face survivors, including managing the adverse effects of cancer and its treatment, seeking information and support, identifying signs and symptoms of disease progression, and making appropriate lifestyle changes for prevention of recurrence or a second cancer [4–6]. Thus, cancer is often viewed as a chronic illness, much like hypertension or diabetes. With this paradigm shift in cancer care, survivors and healthcare providers need to consider that now care is long term, extending beyond the acute phase. This kind of long-term planning requires an ongoing collaborative relationship between survivors and healthcare providers instead of an acute, prescriptive relationship [7].

In order to meet the demand for a new model of cancer care, the Chronic Care Model (CCM) has been suggested [7]. The CCM, which was developed in the US [8] to help general practitioners shift patients from acute care to chronic care, is now widely adopted in various settings [7]. It encourages high-quality care with the following six elements: self-management support, delivery system design, decision support, clinical information systems, healthcare organization, and community resources [8,9]. The goal of CCM-based healthcare is to create an informed, activated patient interacting with a prepared, proactive practice team, resulting in productive encounters and improved outcomes [9–12]. In particular, the “self-management support” element of the CCM enhances patients’ confidence (i.e., self-efficacy) and skills, helping them self-manage their illness [13].

The ability to collect valid data is crucial in evaluating the public-health impact of new initiatives [14]. Glasgow and colleagues [15] developed the Patient Assessment of Chronic Illness Care (PACIC) to assess the quality of patient-centered care for chronic illness consistent with the CCM. The PACIC assesses the receipt of CCM-based chronic care, which emphasizes self-management support (e.g., collaborative goal-setting, problem-solving, and follow-up) and planned, proactive, and population-based care [15]. It has been used to evaluate the delivery of CCM-based intervention for a variety of chronic illness conditions, including diabetes, osteoarthritis, asthma, hypertension, and chronic obstructive pulmonary disease [16–21]. Several studies have demonstrated that the PACIC is an acceptable, valid, and reliable instrument [14,15,22–25], and it is associated with measures of self-management behavior [16,20], self-efficacy [24,26], and health-related quality of life (HRQOL) [27,28].

There are several well-developed tools to assess cancer patients’ needs, such as the Cancer Rehabilitation Evaluation System (CARES) [29], the Comprehensive Needs Assessment Tool in cancer (CNAT) [30], the Need Evaluation Questionnaire (NEQ) [31,32], and the Supportive Care Needs Survey (SCNS) [33]. However, these tools have limitations with regards assessment of the quality of chronic-care delivery. The PACIC is the only tool mentioned in the literature that measures patients’ assessment of chronic-illness care received under the CCM. However, a validation study on the PACIC has not yet been conducted among cancer survivors. In Korea, the PACIC was translated and validated in 2015 [34], but only among patients with hypertension or diabetes. Here, we present data on the psychometric properties of the PACIC among cancer survivors. We examine acceptability, construct validity, and reliability, suggesting the instrument as a potential measure of delivery of CCM-based long-term survivorship care in Korea.

## Methods

### Study design

This study is a secondary analysis using data from a validation study of Cancer Survivors’ Self-Efficacy Scale (CSSES) among 204 patients who had completed their primary cancer treatment [35]. Original data were collected after approval from the Institutional Review Board of Inha University Hospital (No. INHAUH-2017-04-025), and data access and analysis were additionally approved by the same institution for this study (No. INHAUH-2020-05-033).

### Participants and procedures

We recruited 204 study participants from a university-based hospital in Korea using the convenience sampling approach. Participants were: 1) at least 18 years old; 2) those diagnosed with any type of cancer and who had completed their primary treatment (surgery, chemotherapy, or radiation); and 3) those able to read and write Korean. Subjects with evidence of metastasis and/or recurrence, or those undergoing cancer treatment were excluded.

Data were collected from June to July 2017 at an outpatient clinic at Inha University Hospital. Two trained researchers selected eligible patients by visiting potential enrollees in the outpatient department and checking their electronic medical records. During visits, the researchers explained the study, invited participation, and asked for consent to participate. After accepted participants voluntarily signed an informed consent form, the researchers distributed the questionnaire, which took about 20 minutes to complete. Detailed information about recruitment and procedures are described elsewhere [35].

### Sample-size calculation

Based on factorial validity, we chose a sample-size of 200, which is generally acceptable for a confirmatory factor analysis (CFA) [36]. Thus, we used original data (N = 204) [35].

### PACIC and translation process

The original version of PACIC [15] was developed for patients with chronic diseases, that is, hypertension, arthritis, depression, diabetes, asthma, and chronic pain conditions. It demonstrated for a good facial, construct, and concurrent validity [15]. Internal consistency, that is, the Cronbach’s alpha was.93 for the overall PACIC and the item range was.77 to.90 [15]. The PACIC includes 20 items, comprising five subscales: Patient Activation (items 1–3); Delivery System Design/Decision Support (items 4–6); Goal Setting/Tailoring (items 7–11); Problem Solving/Contextual (items 12–15); and Follow-up/Coordination (items 16–20) [15]. It uses a 5-point Likert scale ranging from 1 (“never”) to 5 (“always”), with higher scores indicating better quality of chronic care from the patient’s perspective. Each subscale is scored by averaging items completed within the scale, and the overall PACIC score is the average across all 20 items (Table 2).

In Korea, Kim [34] translated the PACIC into Korean (K-PACIC) using the method recommended by the World Health Organization (WHO) for translation and adaptation of instruments to ensure cross-cultural validity. The process involved a forward–backward translation, discussion by a panel of experts in the field, a back-translation, an original author’s review, and a reconciliation. The K-PACIC was validated with patients with hypertension and diabetes, and it showed good validity and reliability. Internal consistency as represented by Cronbach’s alpha was.93 [34]. We used the K-PACIC in this study with Kim’s permission. After reviewing the K-PACIC items, we revised item 19 (from “Told how my visits with other types of doctors, such as an eye doctor or surgeon, helped my treatment” to “Told how my visits with other types of doctors, such as a psychiatrist or rehabilitation physician, helped my treatment”), reflecting the oncology care setting.

### Measures

#### Cancer Survivors’ Self-Efficacy Scale

The 11 items of the Cancer Survivors’ Self-Efficacy Scale (CSSES) were developed by Foster and colleagues in the United Kingdom (UK) to measure self-efficacy for self-management behaviors in cancer survivors [6]. Each item had 10 response categories with scores ranging from 1 (“not at all”) to 10 (“totally confident”)—higher scores indicating higher self-efficacy. The Korean version of the CSSES (CSSES-K) has10 items and consists of two subscales, including self-efficacy for managing health problems (five items) and self-efficacy for seeking help and support (five items). It has been validated and has shown good reliability [35]. In the present study, the Cronbach’s alpha was.92.

#### HRQOL

The RAND 36-item Short-Form Health Survey (SF-36) is a reliable and validated instrument for measuring HRQOL [37]. It consists of 36 items, eight health concepts (physical functioning, bodily pain, role limitations due to physical health problems, role limitations due to personal or emotional problems, emotional well-being, social functioning, energy/fatigue, and general health perceptions), and a single item (perceived change in health). Total scores range from 0 to 100, with higher scores indicating better quality of life. In this study, SF-36 was translated according to the guidelines provided by RAND [38]. The Cronbach’s alpha was.93.

### Statistical analysis

We assessed the psychometric properties of the K-PACIC among cancer survivors (K-PACIC-CS) in terms of acceptability, construct validity, and reliability. We examined acceptability through analysis of data quality using descriptive statistics, including mean and standard deviation, percentage of missing values, and percentage of floor and ceiling effects. The floor and ceiling effects refer to the percentage of respondents using the most extreme (upper or lower) response categories. We considered a frequency less than 30% acceptable [39].

We evaluated the construct validity of the K-PACIC-CS by verifying factorial structure using the CFA and testing convergent validity. We performed the CFA to confirm the factor structure proposed by Glasgow and colleagues [15]. In order to evaluate the model fit, we used the following indices: the comparative fit index (CFI); the normed fit index (NFI); the Tucker Lewis index (TLI); and the root-mean-square error of approximation (RMSEA). If the CFI, NFI, and TLI were greater than.9 and the RMSEA was.05–.08, the results were deemed acceptable [40,41].

In order to test convergent validity, we used Pearson correlation coefficients. We hypothesized that there would be significant positive correlations between the K-PACIC-CS score and the scores of CSSES-K and SF-36. Here, cancer survivors’ self-efficacy refers to self-efficacy for self-management behaviors after cancer treatment. According to mapping of the PACIC subscales onto the CCM elements [42], it is conceptually sound to use the PACIC as a measure of self-management support in care quality assessments. Self-efficacy is an important outcome of self-management support intervention [43] and was found to be significantly associated with a patient’s perceived self-management support [24,26]. Thus, we expected that the K-PACIC-CS score would be positively correlated with the CSSES-K score.

In order to evaluate reliability, we assessed internal consistency by calculating Cronbach’s alpha, considering a value greater than.70 acceptable [44]. In addition, we used descriptive statistics to report participant characteristics and conducted an independent t-test and one-way ANOVA to examine the difference in the K-PACIC-CS scores by participant characteristics. We performed all analyses using the SPSS 25.0 and the AMOS program (SPSS Inc, Chicago, Illinois).

## Results

### Participant characteristics and the K-PACIC-CS scores

Among 997 potentially eligible participants, 475 did not complete their treatment and 318 refused to participate. Eventually, 204 subjects participated in this study. Table 1 shows participant characteristics and the difference in the K-PACIC-CS scores. The mean age of participants was 54.2 (SD = 11.1) years. Majority of our sample was women (87.7%) and most common types of cancer were breast (75.1%) and gastrointestinal (10.5%) cancers. Almost all (96.6%) had undergone surgery, 66.7% had undergone radiation therapy, and 70.1% had undergone chemotherapy.

**Table 1:** Participant characteristics and the K-PACIC-CS scores.

Characteristics		n (%)	Mean (SD)	t or F	*p*
Age (years)					
< 50		70 (34.3)	3.29 (0.94)	1.55	.216
50–64		96 (47.1)	3.07 (0.86)		
≥ 65		38 (18.6)	3.04 (0.88)		
Gender					
Men		25 (12.3)	3.60 (0.95)	2.79	.006
Women		179 (87.7)	3.08 (0.87)		
Marital status					
Married		151 (74.0)	3.13 (0.92)	-.138	.891
Widowed/divorced/single		53 (26.0)	3.15 (0.83)		
Education level					
< High school graduation		53 (26.0)	2.99 (0.78)	-1.43	.154
≥ High school graduation		151 (74.0)	3.19 (0.93)		
Employment status					
Employed		71 (34.8)	3.27 (0.94)	1.50	.455
Unemployed		133 (65.2)	3.07 (0.86)		
ECOG PS					
0		123 (60.3)	3.24 (0.93)	1.92	.148
1		52 (25.5)	2.99 (0.81)		
2–3		29 (14.2)	2.98 (0.86)		
Number of cancers					
1		181	3.17 (0.90)	1.42	.157
2 or more		22	2.88 (0.80)		
Cancer type^a^ (n = 181)					
Breast		136 (75.1)	3.15 (0.84)	3.34	.021
Thyroid		17 (9.4)	2.78 (1.17)		
Gastrointestinal		19 (10.5)	3.32 (0.93)		
Other^b^		9 (5.0)	3.90 (0.90)		
Cancer stage					
0 & 1		42 (20.6)	2.90 (0.90)	3.25	.041
2		69 (33.8)	3.09 (0.81)		
3		74 (36.3)	3.32 (0.93)		
Treatment experience					
Surgery	Yes	197 (96.6)	3.10 (0.88)	3.28	.001
	No	7 (3.40)	4.20 (0.66)		
Radiation therapy	Yes	136 (66.7)	3.12 (0.86)	0.33	.742
	No	68 (33.3)	3.17 (0.96)		
Chemotherapy	Yes	143 (70.1)	3.19 (0.89)	-1.33	.185
	No	61 (29.9)	3.01 (0.90)		
Months since completion of treatment					
< 12		64 (31.5)	3.39 (0.89)	6.48	.002
12–35		58 (28.6)	3.23 (0.82)		
≥ 36		81 (39.9)	2.88 (0.89)		

^a^Participants with a single cancer.

^b^Other includes hematological, gynecological, and prostate cancer.

ECOG PS = Eastern Cooperative Oncology Group performance status; K-PACIC-CS = Korean version of the Patient Assessment of Chronic Illness Care for cancer survivors.

Men reported significantly higher K-PACIC-CS scores than women (*p* = .006); thyroid cancer survivors had the lowest K-PACIC-CS scores (*p* = .021); participants diagnosed with Stage 3 showed significantly higher K-PACIC-CS scores as compared to those with Stages 0, 1, or 2. Participants whose treatment had been completed less than 12 months ago revealed significantly higher K-PACIC-CS scores than those whose treatment had been completed more than 36 months ago (*p* = .002) (Table 1).

### Acceptability

Table 2 presents descriptive statistics of the K-PACIC-CS. The missing rate was only 0.5%, and only Item 5 had a missing value.

**Table 2:** Descriptive statistics of the K-PACIC-CS (N = 204).

Item	Mean (SD)	Floor Effect	Ceiling Effect
		n (%)	
**Patient Activation**	3.82 (1.04)	3 (1.5)	43 (21.1)
Q1	3.97 (1.15)	8 (3.9)	84 (41.2)
Q2	3.76 (1.20)	10 (4.9)	65 (31.9)
Q3	3.75 (1.33)	19 (9.3)	76 (37.3)
**Delivery System Design/Decision Support**	3.74 (0.97)	0 (0.0)	31 (15.2)
Q4	3.25 (1.37)	30 (14.7)	41 (20.1)
Q5	4.07 (1.02)	7 (3.4)	81 (39.7)
Q6	3.91 (1.11)	11 (5.4)	68 (33.3)
**Goal Setting/Tailoring**	3.03 (1.08)	8 (3.9)	7 (3.4)
Q7	3.31 (1.35)	29 (14.2)	45 (22.1)
Q8	3.49 (1.23)	18 (8.8)	47 (23.0)
Q9	2.65 (1.49)	64 (31.4)	34 (16.7)
Q10	3.01 (1.43)	44 (21.6)	35 (17.2)
Q11	2.68 (1.40)	59 (28.9)	23 (11.3)
**Problem Solving/Contextual**	3.29 (1.07)	8 (3.9)	15 (7.4)
Q12	3.37 (1.32)	28 (13.7)	43 (21.1)
Q13	3.40 (1.28)	25 (12.3)	39 (19.1)
Q14	3.43 (1.22)	19 (9.3)	36 (17.6)
Q15	2.95 (1.37)	42 (20.6)	29 (14.2)
**Follow-up/Coordination**	2.36 (1.10)	34 (16.7)	3 (1.5)
Q16	2.59 (1.40)	55 (27.0)	30 (14.7)
Q17	2.24 (1.31)	83 (40.7)	14 (6.9)
Q18	2.26 (1.39)	86 (42.2)	20 (9.8)
Q19	2.49 (1.46)	79 (38.7)	23 (11.3)
Q20	2.23 (1.34)	89 (43.6)	16 (7.8)
K-PACIC-CS total	3.14 (0.89)		

K-PACIC-CS = Korean version of the Patient Assessment of Chronic Illness Care for cancer survivors.

### Validity

#### Factorial validity: CFA

We performed the CFA according to the hypothesized factor structure identified by Glasgow and colleagues [15]. Given the large modification index, we added one correlation of error covariance. Among the four indices for model fit, our data fit well for the CFI, the TLI, and the RMSEA, but not for the NFI (Table 3).

**Table 3:** Model fit of confirmatory factor analysis of the K-PACIC-CS (N = 204).

Fit Index				Relative Fit Index		Absolute Fit Index	
	χ^2^	Df	*p*	CFI	NFI	TLI	RMSEA
K-PACIC-CS	336.24	159	< .001	.93	.87	.91	.07
Criterion for Good Fit				≥.90	≥.90	≥.90	<.08

K-PACIC-CS = Korean version of the Patient Assessment of Chronic Illness Care for cancer survivors.

#### Convergent validity

The K-PACIC-CS correlated significantly and positively with both the CSSES-K (r = .25, *p* < .001) and SF-36 (r = .25, *p* < .001).

### Reliability

#### Internal consistency

Cronbach’s alpha of the K-PACIC-CS was.94 for the total scale; the results for the subscales are as follows: Patient Activation, .80; Delivery System Design/Decision Support, .76; Goal Setting/Tailoring, .84; Problem Solving/Contextual, .84; and Follow-up/Coordination, .86.

## Discussion

The PACIC provides a brief patient assessment of the extent to which chronically ill patients report receiving care that is consistent with the CCM [15]. As we consider the application of CCM to Korean cancer survivors, a psychometric evaluation of the K-PACIC-CS is timely and appropriate. Our analyses demonstrated a good fit of the proposed PACIC factorial structure, reasonable convergent validity, and excellent reliability. To the best of our knowledge, this is the first validation study of PACIC for a cancer population, and our findings may contribute to the implementation of CCM-based cancer survivorship care.

The mean scores on the K-PACIC-CS in our sample were somewhat higher than those reported in non-cancer populations in the US [15] and Europe [21,22,24,45] because of the high mean scores of the Patient Activation (mean = 3.82) and Delivery System Design/Decision Support (mean = 3.74) subscales, which may be explained by the nature of the oncology treatment environment. A multidisciplinary team (MDT) approach with team decision-making is currently the standard of cancer treatment [45], so cancer patients seem to participate more frequently in treatment decisions compared to other chronic patient populations.

Regarding floor and ceiling effects, the results vary greatly. Although the PACIC suffers overall from floor effects versus ceiling effects [14,21,24,25,45,46], that was not evident in our study. We found similar results for floor effects (range, 3.9%– 43.6%; > 30% for five items) and ceiling effects (range, 6.9%– 41.2%; > 30% for five items). Consistent with previous findings [14,21,45–47], we found a floor effect in the Follow-up/Coordination subscale. In particular, Item 17 (“Encouraged to attend program in the community that could help me”) had a higher frequency in the lowest response category in several studies (30.7%– 92.5%) [14,21,45–47], including ours (40.7%). This suggests that the current cancer care delivery systems in the acute and primary care settings are not well-coordinated.

We also observed notable floor effects (38.7%– 43.6%) in other items (referral to dietician, consultation, and communication related to consultation), which we did not expect because Korea has adopted the MDT approach [48], which involves consultations and referrals. Current MDT practice, however, may be focused on the initial treatment phase, not the survivorship period. According to Maeng and colleagues [48], the MDT focus in Korea is decision-making for further treatment planning and prediction of prognosis. Therefore, we should be careful about interpreting a high floor effect in the Follow-up/Coordination subscale when administering the K-PACIC-CS to long-term cancer survivors.

In addition, Item 9 (“Given a copy of my treatment plan.”) showed a high floor effect (31.4%). This contrast with the US value may be related to differences in the US and Korean healthcare systems. In Korea, as opposed to the US, the treatment plan developed by the physician is not shared with the patient, who is not even given a copy of it. Instead, patients are given materials that include information about the next visit and test schedules. Many of our participant responses to Item 9 might have related to those materials rather than the treatment plan. In order to clarify the meaning of Item 9, we recommend using the term ‘자료’ (material) instead of ‘사본’ (copy) when it is translated into Korean.

The CFA analysis showed a good fit with three K-PACIC-CS indices, which supports the five-factor structure predetermined by Glasgow and colleagues [15], and is consistent with the Danish [14] and Dutch studies [23]. Three CFA analyses [18,45,49], however, failed to verify the predetermined five-factor PACIC structure. Other research using the EFA analysis identified a three-factor [24,25] or two-factor structure [24,46,47] (even Gugiu and colleagues [18] suggested the PACIC could be a unidimensional tool). These differences may reflect methodological differences, such as the CFA versus the EFA, study sample differences (e.g., patients with diabetes [24], cardiovascular disease [23], osteoarthritis [19], chronic obstructive respiratory disease [21], or a mixed group [20]), or health system differences (e.g., in the US [15], the UK [45], France [46], the Netherlands [21], Denmark [14], Finland [24], Slovenia [47], and Malaysia [25]).

The K-PACIC-CS had a significant relationship with the CSSES-K and the SF-36 as hypothesized, confirming convergent validity. The correlation coefficient (.25 for each), however, was relatively weak compared to earlier studies [15,47]. Generally, r≥.4 is regarded as optimal [50]. Previous researchers have used the Patient Self-Activation Scale [15]and the EUROPEP instrument to assess patient satisfaction with medical care in the primary care setting [47]. Compared to those tools, self-efficacy for self-management and the HRQOL seem to be more comprehensive. This is a limitation of secondary analysis studies; therefore, further investigation is required.

We provided data of factors associated with the K-PACIC-CS, which could verify known groups validity. Due to lack of prior evidence, we could not determine known groups. We suggest cancer stage (early *vs* advanced) and time since completion of treatment (short-term survivorship phase *vs* long-term) as a known group. In the current study, survivors with advanced stage cancer showed higher K-PACIC-CS scores than those in early stages. It is possible that patients with advanced cancer are more likely to be involved in decision-making, problem-solving, goal-setting and adjustment, and follow-up. Regarding time since completion of treatment, survivors in the short-term survivorship phase have more frequent visits scheduled than those in long-term survivorship phase, yielding higher K-PACIC-CS scores.

## Study limitations

First, our sample consisted mostly of women treated for breast cancer, restricting the generalizability of our findings. Stratified sampling would have been the best choice for this study. Future research using the K-PACIC-CS should report psychometric properties such as Cronbach’s alpha. Second, as this was a secondary analysis study, we tested internal consistency with Cronbach’s alpha only in terms of reliability; there is a need to also examine reproducibility such as test-retest reliability. Third, we did not evaluate responsiveness, which could contribute to a more psychometrically sound tool for testing the effects of a CCM-based intervention. Lastly, the low missing-data rate of this study (0.5%) suggests the possibility that research assistants might have helped participants respond if they faced difficulties understanding items (a limitation following from secondary analysis).

## Conclusions

The psychometric properties of the K-PACIC-CS among cancer survivors are satisfactory and suggest a promising way to evaluate cancer survivors’ perspective of a CCM-based survivorship care.
